# Pharmacological strategies to minimize or avoid neuromuscular blocking agents during general anesthesia: a comprehensive approach from airway management to surgical requirements

**DOI:** 10.3389/fphar.2026.1781953

**Published:** 2026-03-12

**Authors:** Fang-Qin Zhan, Rui Guo

**Affiliations:** 1 Department of Anesthesiology and Perioperative Medicine, Mengchao Hepatobiliary Hospital of Fujian Medical University, Fuzhou, Fujian, China; 2 Department of Anesthesiology, First Affiliated Hospital of Gannan Medical University, Ganzhou, Jiangxi, China

**Keywords:** airway management, general anesthesia, neuromuscular blocking agents, pharmacological strategies, sugammadex, surgical exposure

## Abstract

Neuromuscular blocking agents (NMBAs) are commonly used in general anesthesia and are widely applied in airway management and surgical requirements. However, the traditional use of NMBAs in anesthetic strategies has exposed potential issues, particularly residual neuromuscular blockade, which is associated with respiratory complications, delayed recovery, and prolonged hospitalization. In recent years, strategies aimed at minimizing or avoiding NMBAs have gained attention, especially in balancing airway management with surgical exposure demands during general anesthesia. This review was designed as a structured narrative review rather than a formal systematic review or meta-analysis. This review summarizes the pharmacological strategies for minimizing or avoiding NMBAs, with a focus on distinguishing between NMBA-free anesthesia, which completely avoids NMBAs, and NMBA-sparing anesthesia, which uses low doses or phased administration to minimize their use. Through the combination of other anesthetic agents and depth modulation, strategies for NMBA-free and NMBA-sparing have shown positive results in certain patients and surgeries, particularly in day surgery and fast-track recovery pathways. Nevertheless, this strategy is not suitable for all patients and surgeries, and individualized anesthetic management remains key to its successful implementation. Furthermore, with the introduction of Sugammadex, the rapid reversal of neuromuscular blockade provides assurance for minimizing or avoiding the use of NMBAs. Future research should focus on optimizing drug combinations, verifying non-inferiority, and applying these strategies to specific patient populations, further promoting their clinical adoption.

## 1 Introduction

In general anesthesia, neuromuscular blocking agents (NMBAs) are crucial for ensuring airway management and surgical requirements. However, their use is associated with potential issues, including residual neuromuscular blockade (RNMB), which has been reported in up to approximately 65% of patients at tracheal extubation in some multicentre studies, as well as postoperative pulmonary complications (PPCs) and delayed postoperative recovery (Feltracco et al., 2016; Raval et al., 2020; Bijkerk et al., 2025; Brull and Murphy, 2010; Murphy and Brull, 2010; Kirmeier et al., 2019). The POPULAR study, for instance, found that NMBA use was associated with an increased incidence of PPCs (ORadj 1.86, 95% CI 1.53–2.26) after adjustment for confounders (Kirmeier et al., 2019).

Not all instances of airway management or surgical procedures necessarily require complete muscle relaxation. To address this, two distinct pharmacological strategies have emerged: NMBA-free anesthesia and NMBA-sparing anesthesia. NMBA-free anesthesia involves the complete avoidance of NMBAs. In contrast, NMBA-sparing anesthesia refers to the use of reduced doses or phased administration of NMBAs, minimizing their use while still maintaining some degree of muscle relaxation for more complex procedures. Unless otherwise specified, the term “minimizing or avoiding NMBAs” is used in this review as an umbrella term encompassing both NMBA-free and NMBA-sparing approaches.

The need to minimize or avoid NMBAs is particularly evident in specific clinical contexts, such as during intraoperative electrophysiological monitoring (Sloan, 2013), intraoperative wake-up procedures (Kulikov and Lubnin, 2018; Fukaya et al., 2001), or in patients with neuromuscular disorders like Myasthenia Gravis (Radkowski et al., 2024; Hien et al., 2023). Although the introduction of sugammadex has significantly improved the reversal of NMBAs, its limitations—including cost, variable availability, and suboptimal reversal under deep blockade—mean it has not eliminated all NMBA-related concerns (Honing et al., 2019; Kotake et al., 2013; Renew and Linn, 2025; Ito et al., 2019). Importantly, the safe implementation of these strategies relies on objective quantitative neuromuscular monitoring (e.g., TOF monitoring) to minimize RNMB risk.

In selected settings, strategies aimed at minimizing or avoiding NMBAs may help reduce postoperative respiratory complications and facilitate recovery (Cammu, 2020; Frenkel and Lien, 2024; Kiekkas et al., 2014), particularly showing significant advantages in day surgery and fast-track recovery protocols. However, this approach is not universally applicable. Factors such as airway management, surgical exposure requirements, and the patient’s physiological condition critically influence the choice of anesthetic strategy (Fuchs-Buder et al., 2023). Therefore, this review aims to summarize the pharmacological strategies for minimizing or avoiding NMBAs, exploring their application, advantages, and potential risks across different stages of anesthesia.

## 2 Methods

### 2.1 Literature search and screening process

This review was designed as a structured narrative review informed by a multi-database literature search, with the aim of providing a comprehensive and clinically oriented synthesis of pharmacological strategies for minimizing or avoiding neuromuscular blocking agents across diverse perioperative contexts. It was not conducted as a formal systematic review or meta-analysis.

The literature search was conducted independently by two authors (Fang-qin Zhan and Rui Guo), using multiple databases, including PubMed, Embase, Cochrane Library, Web of Science, and Google Scholar. The search strategy combined Medical Subject Headings (MeSH) terms and free-text keywords, including but not limited to “neuromuscular blocking agents,” “muscle relaxants,” “airway management,” “general anesthesia,” “pharmacological strategies,” “minimization,” “avoidance,” “sugammadex,” and “residual neuromuscular blockade.” Reference lists of relevant articles were also manually screened to identify additional studies. The search covered studies published between January 2001 and November 2025.

Eligibility criteria: Studies were included if they involved human subjects undergoing general anesthesia and (1) addressed pharmacological strategies intended to minimize or avoid NMBAs for airway management and/or surgical requirements, or (2) reported clinically relevant outcomes (e.g., intubating conditions, residual neuromuscular blockade, postoperative respiratory complications, or recovery-related outcomes). We excluded non-English publications, animal or *in vitro* studies, conference abstracts without full text, and articles lacking direct clinical relevance to NMBA-free or NMBA-sparing approaches.

**FIGURE 1:**
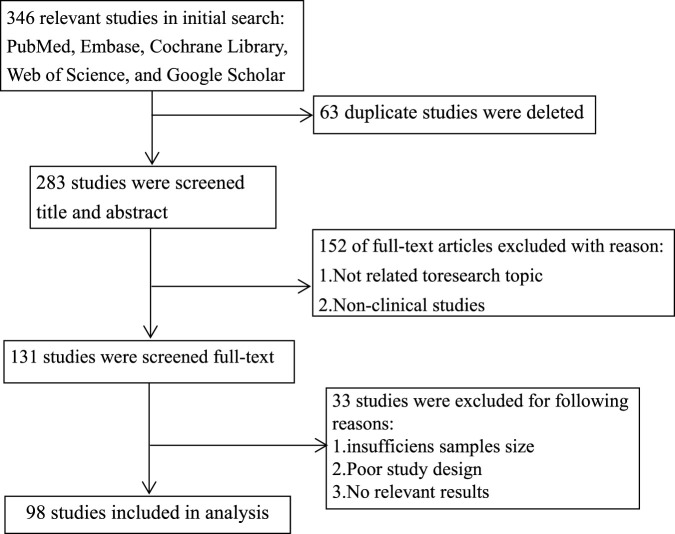
Literature search and screening process.

A total of 346 records were initially identified through database searching. After removal of 63 duplicates, 283 records remained for title and abstract screening. Subsequently, 131 full-text articles were assessed for eligibility, and 98 studies met the eligibility criteria and were included in the final qualitative synthesis. The search and selection process is presented using a PRISMA-style flow diagram to enhance transparency (Figure 1). The databases listed in Figure 1 were cross-checked to ensure consistency with the Methods section.

### 2.2 Literature review and quality considerations

All eligible articles were independently reviewed by the two authors (Fang-qin Zhan and Rui Guo). Each study was assessed for relevance to the review topic and for key methodological considerations, including study design, patient population, pharmacological interventions, and clinical applicability to anesthesia practice. Studies that did not meet the eligibility criteria or lacked sufficient relevance were excluded. Formal risk-of-bias assessment tools were not applied, as the primary aim of this structured narrative review was to provide a conceptual and practical overview rather than a quantitative comparison of effect sizes. In addition, no formal evidence grading framework (e.g., GRADE) was used.

### 2.3 Discrepancy resolution

Any discrepancies regarding study inclusion or data interpretation were resolved through discussion and consensus between the two authors. In cases of disagreement, the full text was re-evaluated jointly until a final decision was reached.

## 3 Pharmacological basis for minimizing or avoiding NMBAs

In general anesthesia, strategies for minimizing or avoiding NMBAs rely on the pharmacological properties of various drugs. The mechanisms of action, dose-effect relationships, and synergistic effects of drug combinations are crucial to achieving this goal. The following sections highlight the roles of several commonly used drugs in minimizing or avoiding NMBAs.

### 3.1 Inhaled anesthetics

Inhaled anesthetics (e.g., sevoflurane, desflurane, isoflurane) are commonly used agents in general anesthesia. These drugs induce loss of consciousness, effectively suppress airway reflexes, and reduce muscle tone, which provides favorable conditions for airway management. By acting on the central nervous system, inhaled anesthetics decrease anesthesia depth, reduce sympathetic responses, and minimize airway reflexes (He et al., 2014). This is particularly beneficial during airway manipulations such as intubation and laryngeal mask airway (LMA) insertion, thereby minimizing the need for NMBAs.

For example, sevoflurane effectively suppresses pharyngeal reflexes and decreases muscle tone at appropriate anesthesia depths (Joo et al., 2001). By adjusting anesthesia depth, reliance on traditional NMBAs can be reduced, especially for airway management requiring minimal muscle relaxation (Min et al., 2007). However, the use of inhaled anesthetics alone is associated with certain risks, such as intraoperative hemodynamic instability and postoperative nausea and vomiting (Wappler et al., 2003). Therefore, inhaled anesthetics are often combined with other drugs to achieve optimal pharmacological effects.

### 3.2 Opioids

Opioids, particularly remifentanil and fentanyl, are commonly used for analgesia in anesthesia. Opioids act on the central nervous system’s opioid receptors to suppress airway reflexes, reduce stress responses during intubation, and improve intubation success rates (Joo et al., 2001).

Opioids are particularly effective in providing analgesia and alleviating stress responses during anesthetic induction and airway management. Studies have shown that appropriate opioid administration can inhibit pharyngeal reflexes and sympathetic responses without full reliance on NMBAs, particularly when using remifentanil (Min et al., 2007; He et al., 2009; Demirkaya et al., 2012; Ming et al., 2025; Soulard et al., 2009; Akan et al., 2025). It significantly reduces laryngospasm and airway reflexes during intubation. However, opioid use is also associated with adverse effects such as hypotension and chest rigidity, which are more pronounced when NMBAs are not used. Therefore, opioids need to be combined with other drugs to reduce side effects and enhance airway management safety and efficacy.

### 3.3 Propofol or ciprofol

Propofol is a commonly used intravenous anesthetic known for its rapid onset and rapid recovery. Propofol acts on the central nervous system to provide adequate anesthetic depth while minimizing reliance on NMBAs (Demirkaya et al., 2012; Lerman et al., 2009; Gao et al., 2025; Siddik-Sayyid et al., 2011; Liao et al., 2025). It effectively suppresses airway reflexes, reduces stress responses during intubation, and is particularly useful for airway management that requires minimal muscle relaxation.

Although propofol has significant anesthetic effects, it may cause hypotension, which requires careful dose adjustment. While ciprofol, a newer anesthetic with similar pharmacological properties, shares many of the advantages of propofol, it may offer some improvements in terms of reduced hypotension and a more stable hemodynamic profile in certain patients (Guo et al., 2024; Hung et al., 2023). Both agents are effective adjuncts in minimizing the use of NMBAs (Siddik-Sayyid et al., 2011; Liao et al., 2025; Guo et al., 2024), as they provide precise control over anesthesia depth and beneficial effects on airway management.

### 3.4 Dexmedetomidine

Dexmedetomidine, an α_2_-adrenergic agonist, plays an important role in strategies to reduce or avoid NMBAs. Dexmedetomidine acts on central α_2_ receptors to provide potent sedative and analgesic effects without significantly depressing respiration. Its sedative effect is similar to natural sleep, and it reduces sympathetic activity, lowers blood pressure, and alleviates intubation stress responses.

In general anesthesia, when dexmedetomidine is used in combination with opioids or inhaled anesthetics, it significantly enhances the suppression of airway reflexes (He et al., 2014; Wei et al., 2017; Zeng et al., 2025). Research has shown that the combination of opioids and dexmedetomidine can reduce stress responses during airway manipulation without increasing opioid dosage, thus achieving safer airway management (Zeng et al., 2025).

### 3.5 Local anesthetics (airway surface anesthesia)

Local anesthetics, such as lidocaine or dibucaine spray and topical airway anesthesia, are widely used in airway management to reduce airway reflexes and alleviate stress responses during intubation, thereby minimizing the need for NMBAs, particularly in cases involving relatively simple airway manipulation (Fjærestad et al., 2025; Lv et al., 2020; Takaenoki et al., 2016). Airway surface anesthesia offers the advantage of rapid onset and relatively fewer side effects, making it an effective adjunct to general anesthesia for airway management. For a comparison of the pharmacological properties, advantages, and limitations of various agents, see Table 1.

**TABLE 1:** Comparison of pharmacological agents used to reduce or avoid neuromuscular blockade.

Drug type	Mechanism of action	Evidence level	Patient population	Advantages	Limitations	References
Inhaled Anesthetics (e.g., Sevoflurane)	Suppresses airway reflexes and reduces muscle tone through CNS action	RCT	Adult, Pediatric	Reduces airway reflexes, facilitates minimal muscle relaxation for airway management	Potential hemodynamic instability, postoperative nausea and vomiting	He et al. (2014), Joo et al. (2001), Min et al. (2007), He et al. (2009)
Opioids	Acts on opioid receptors in CNS to suppress airway reflexes and provide analgesia	RCT	Adult, Pediatric	Strong analgesic effects, reduces stress response during intubation	May cause chest rigidity, hypotension	Joo et al. (2001), He et al. (2009), Demirkaya et al. (2012), Ming et al. (2025), Soulard et al. (2009), Akan et al. (2025)
Propofol (or ciprofol)	Acts on CNS to provide anesthetic depth and reduce reliance on NMBAs	RCT	Adult, Pediatric	Rapid onset and recovery, effective for airway management with minimal muscle relaxation	hypotension	Demirkaya et al. (2012), Lerman et al. (2009), Gao et al. (2025), Siddik-Sayyid et al. (2011), Liao et al. (2025), Ide et al. (2014), Seangrung et al. (2021)
Dexmedetomidine	Provides sedation and analgesia via central α_2_ receptor action, without significant respiratory depression	RCT Observational	Adult	Sedation similar to natural sleep, few side effects	Can cause hypotension with prolonged use, slow postoperative recovery	He et al. (2014), Gao et al. (2025), Wei et al. (2017), Zeng et al. (2025), Seangrung et al. (2021), Gupta et al. (2023), De Cassai et al. (2021)
Lidocaine (intravenous)	Inhibition of laryngeal reflexes and anti-sympathetic nervous system effects	RCT	Adult	Reduction of intubation reflexes and cardiovascular responses	May cause central nervous system toxicity and cardiovascular toxicity	Hanci et al. (2010), Jabbour-Khoury et al. (2003), Hashemian et al. (2018)
Local Anesthetics (Airway surface anesthesia)	Local anesthetic effects on airway to suppress reflexes and provide analgesia	RCT	Adult (older people)	Reduces dependence on neuromuscular blocking agents, fast onset	Dependent on dosage, application site, and practitioner technique	Fjærestad et al. (2025), Lv et al. (2020), Takaenoki et al. (2016)

Abbreviations: CNS, central nervous system; RCT, randomized controlled trial.

## 4 Strategies for minimizing or avoiding NMBAs use in airway management

Airway management is a core component of general anesthesia, particularly during tracheal intubation or supraglottic airway device insertion. Traditionally, airway management has relied on NMBAs to facilitate glottic exposure and prevent reflexive spasms. However, with advancements in anesthetic pharmacology, a new trend has emerged where drug combinations and depth of anesthesia are adjusted to reduce or eliminate the need for NMBAs in airway management.

### 4.1 Pharmacological strategies in tracheal intubation

Tracheal intubation is one of the most common and challenging procedures in general anesthesia, typically requiring complete muscle relaxation to ensure smooth intubation. The widespread use of video laryngoscopy in recent years has significantly improved the visualization during intubation, thus minimizing the reliance on complete muscle relaxation to some extent (Creutzburg et al., 2025). With advancements in anesthesia, an increasing number of studies suggest that in certain patients and surgeries, a strategy involving NMBA-free or NMBA-sparing approaches may still provide optimal intubation conditions (Lundstrøm et al., 2018). However, the definition of “acceptable intubating conditions” varies across studies, and commonly used scoring systems may not fully capture patient-centred outcomes (e.g., haemodynamic stability, airway trauma, hypoxaemia, or postoperative morbidity), including laryngeal morbidity (Mencke et al., 2003). Therefore, NMBA-free or markedly NMBA-sparing intubation should be considered only in selected patients, such as those without difficult airways, with priority given to first-pass success and overall airway safety (Sneyd and O'Sullivan, 2010). This consideration is particularly important in pediatric patients (Morton, 2009), where a recent systematic review and meta-analysis demonstrated that the use of NMBAs was associated with improved intubating conditions and a lower risk of failed first intubation attempts compared with NMBA-free techniques (Vanlinthout et al., 2020).

#### 4.1.1 Combination of anesthetic drugs

In strategies involving no NMBAs, a combination of propofol, opioids (such as remifentanil or fentanyl), inhalational anesthetics, and dexmedetomidine can effectively attenuate airway reflexes and improve intubation conditions (Joo et al., 2001; Demirkaya et al., 2012; Hanci et al., 2010; Jabbour-Khoury et al., 2003; Lundstrøm et al., 2018; Ovari et al., 2017). Specifically, combining two or more of these drugs, such as propofol and remifentanil, can significantly reduce stress responses during tracheal intubation (Mencke et al., 2014; Blair et al., 2004), such as laryngospasm and coughing (Ide et al., 2014). Dexmedetomidine, with its sedative properties, further suppresses airway reflexes, deepens anesthesia, and reduces the need for muscle relaxants. However, NMBA-free intubation may increase coughing, laryngospasm, and airway trauma in some patients (Mencke et al., 2003), and opioid-based techniques may introduce less predictable respiratory depression (including chest wall rigidity) and apnea. Importantly, much of the available evidence is derived from selected low-risk elective populations and may not be generalizable to patients with predicted difficult airways, obesity, significant aspiration risk, or limited cardiopulmonary reserve. Therefore, careful dosing, appropriate rescue preparedness, and strict patient selection are essential to maintain airway safety and ensure first-pass success.

#### 4.1.2 Airway surface anesthesia

Adequate airway surface anesthesia, such as the application or nebulization of local anesthetics to the larynx and airway prior to intubation or the application of local anesthetic ointment to the tracheal tube, can effectively reduce airway reflexes and minimize the use of NMBAs (Rahmat Ameen Noorazyze et al., 2022; Zhang et al., 2023). Sufficient airway surface anesthesia, especially in surgeries requiring no muscle relaxation but absolute immobility during the procedure (e.g., microsurgical procedures or intracranial aneurysm embolization), can prevent inadvertent emergency events caused by coughing during the operation. Routine use of appropriate NMBAs, in combination with maintaining proper depth of anesthesia, can effectively prevent such events, while sufficient airway surface anesthesia can help reduce the need for muscle relaxants.

Airway surface anesthesia is particularly valuable in patients with predicted difficult airways, obesity, or significant airway obstruction. In such patients, awake intubation under appropriate sedation and analgesia, combined with adequate airway surface anesthesia, represents a safe and effective strategy to maintain spontaneous breathing and ensure airway patency. Once ventilation is confirmed to be adequate, NMBAs may be carefully administered to facilitate intubation if needed. In contrast, for patients without these risk factors, airway surface anesthesia can still serve as an effective adjunct to reduce NMBA requirements during routine intubation.

### 4.2 Pharmacological strategies in laryngeal mask airway (LMA) insertion

Compared to tracheal intubation, LMA insertion relies less on NMBAs, as it does not require complete elimination of vocal cord tension or pharyngeal muscle activity. Therefore, LMA insertion has become an important application for NMBA-free or NMBA-sparing strategies (Totonchi et al., 2022).

In particular, second-generation LMAs (such as the ProSeal LMA) have been optimized in design, providing better airway sealing and incorporating a gastric drainage channel that reduces the risk of gastric content aspiration. Unlike tracheal intubation, LMA insertion is simpler to perform and can be successfully completed with an appropriate depth of anesthesia, especially in a minimal or no muscle relaxant state (Rahmat Ameen Noorazyze et al., 2022; Tang et al., 2023). Its use ensures airway safety while minimizing the need for muscle relaxants.

In low-risk patients and day-case surgeries (Tulgar et al., 2017), the combination of NMBA-free or NMBA-sparing with LMA insertion has been widely applied in general anesthesia, further enhancing surgical safety and accelerating postoperative recovery. These findings primarily apply to carefully selected low-risk patients; in patients with high aspiration risk, significant obesity, or anticipated difficult airway, minimizing or avoiding NMBAs should not compromise airway protection strategies.

## 5 Strategies for minimizing or avoiding NMBAs in surgical requirements

While airway management is a critical component of general anesthesia, in many surgical procedures, the use of NMBAs extends beyond this purpose and is often essential for ensuring adequate surgical field exposure. Sufficient NMBAs use is particularly important in operations requiring extensive surgical access.

### 5.1 The requirement for muscle relaxation in surgical exposure

Surgical exposure refers to the degree of visibility and accessibility of the operative field, which typically relies on the effects of NMBAs. In certain surgeries, especially open/laparoscopic abdominal and open/video-assisted thoracic procedures, a profound degree of muscle relaxation is necessary to achieve optimal surgical exposure and muscle laxity.

### 5.2 Surgical types traditionally considered to require strong neuromuscular blockade

1. Open/Laparoscopic Abdominal Surgery: Both open incisions and laparoscopic pneumoperitoneum have traditionally been managed under complete muscle relaxation. Open surgery necessitates relaxation for optimal surgical field exposure and visceral manipulation, while laparoscopic surgery requires muscle relaxation to counteract intra-abdominal pressure from pneumoperitoneum, preventing reflexive muscle contraction and providing a stable working space for instruments (Díaz-Cambronero et al., 2023). However, for certain simple laparoscopic procedures, such as cholecystectomy or gynecological mass removal, the surgical requirements can be met with minimal or no use of NMBAs, utilizing alternative drug combinations. A study by Sule Ozbilgin et al. (Tulgar et al., 2017) demonstrated that during laparoscopic gynecological surgeries, airway management with the next-generation laryngeal mask airway allowed for satisfactory ventilation and surgical field quality without the need for NMBAs. Nevertheless, the available evidence remains heterogeneous and is often limited by small sample sizes and selected patient populations. Therefore, these findings should not be generalized to all laparoscopic procedures or to patients with higher perioperative risk.
2. Open/Video-Assisted Thoracic Surgery: These procedures have conventionally been performed with complete muscle relaxation to prevent mediastinal shift and paradoxical respiration caused by spontaneous breathing. This ensures stabilization of lung tissue and a motionless surgical field, creating optimal conditions for precise manipulation of critical structures such as the heart, great vessels, or pulmonary hilum.
3. Precision Surgery Requiring Absolute Immobility: Examples include microsurgery and intracranial endovascular interventions. While these procedures do not rely on profound neuromuscular blockade, maintaining a moderate level of muscle relaxation is crucial to effectively prevent accidents such as coughing or movement, thereby serving as a key factor in ensuring procedural safety.

### 5.3 Surgical procedures requiring minimal or avoidance of NMBAs

#### 5.3.1 Surgical procedures requiring intraoperative neurophysiological monitoring

Certain procedures necessitate specific adjustments to neuromuscular blockade management. In neurosurgical or orthopedic surgeries requiring intraoperative neurophysiological monitoring, the use of NMBAs is typically avoided or strictly minimized to prevent interference with the recordings of motor evoked potentials (MEPs) or electromyography (EMG) signals (Jian et al., 2023; Zhang et al., 2022).

#### 5.3.2 Surgical procedures requiring intraoperative awakening

For awake craniotomies requiring cortical mapping, a no- or low-dose NMBAs strategy combined with a LMA is commonly employed (Fukaya et al., 2001). This permits safe airway device removal upon awakening, enabling patient cooperation during functional testing while maintaining airway control during asleep phases.

#### 5.3.3 Application of tubeless techniques in thoracic surgery

In recent years, the application of tubeless techniques in thoracic surgery has gained increasing attention, particularly for low-risk patients undergoing procedures such as pulmonary nodule resection or bullae resection, including in day surgery. Tubeless strategies encompass the avoidance of endotracheal intubation, chest drains, and urinary catheters, with the core principles being safety assurance and accelerated recovery. The tubeless anesthesia approach, often involving a LMA combined with a minimal- or zero-NMBA strategy. This strategy has been associated with significantly shortened postoperative recovery times and a marked reduction in the incidence of postoperative respiratory complications (He et al., 2019; Fabo et al., 2021; Liu et al., 2023).

#### 5.3.4 Patients with neuromuscular disorders

General anesthesia in patients with neuromuscular disorders (Radkowski et al., 2024; Hien et al., 2023), including Guillain-Barré Syndrome, Myasthenia Gravis, Duchenne Muscular Dystrophy, Charcot-Marie-Tooth Disease, and Inflammatory Myopathies, should be carefully managed, with the use of NMBAs minimized or avoided whenever possible, as these conditions may increase sensitivity to muscle relaxants (Fujita et al., 2015; Tagawa et al., 2009).

### 5.4 Phased modulation of NMBAs

During surgery, anesthesiologists can flexibly adjust the use of NMBAs according to specific procedural phases. At the initiation of surgery or during critical stages requiring optimal exposure, a higher degree of muscle relaxation can be maintained to ensure adequate surgical access and facilitate smooth operation. In other phases, particularly during less invasive parts of the procedure or the recovery period, minimizing or avoiding NMBAs use can minimize unnecessary pharmacological dependence and promote faster postoperative recovery. Importantly, when optimal exposure or immobility is critical (e.g., certain laparoscopic/thoracic stages), maintaining adequate neuromuscular blockade may define the safety boundary beyond which further NMBA reduction could increase procedural risk.

### 5.5 Combined use of anesthetic agents and minimizing reliance on NMBAs

The rational combination of anesthetic agents can effectively reduce the need for traditional NMBAs while ensuring adequate anesthetic depth and muscle relaxation during surgery. Sevoflurane, a commonly used inhaled anesthetic, has been demonstrated to provide not only sufficient anesthetic depth at appropriate concentrations but also a certain degree of muscle relaxant effect, and it has a synergistic effect when combined with NMBAs (Kang et al., 2017). Desflurane anaesthesia significantly prolongs the duration of action of rocuronium at 0.9 mg/kg single bolus dose (Maidatsi et al., 2004).

Furthermore, the combined use of propofol and opioids (such as remifentanil) can effectively enhance analgesia, reduce intraoperative stress response, and decrease reliance on NMBAs (Wu et al., 2023). Paek et al. (2009) found that propofol and remifentanil anesthesia for laparoscopic pelvic surgery does not require supplemental muscle relaxants, as no significant differences in cardiorespiratory parameters were observed between groups with and without muscle relaxants.

### 5.6 General anesthesia combined with epidural anesthesia

For some abdominal or pelvic surgeries, the combination of general anesthesia with spinal or epidural anesthesia can reduce the need for NMBAs (Munakata et al., 2004; Sahin et al., 2011; Suzuki et al., 2007). Local anesthetics used in epidural blocks inhibit motor nerve conduction, leading to relaxation of the corresponding muscle groups. This approach is particularly suitable for major open abdominal and pelvic procedures (Hossain et al., 2012). The strategies for minimizing or avoiding NMBAs in airway management and surgical settings are summarized in Table 2.

**TABLE 2:** Strategies for minimizing or avoiding NMBAs in airway management and surgical requirements.

Scenario	Key strategies and methods	Evidence level	References
Airway management			
1. Endotracheal Intubation (without NMBAs)	Drug combinations: sevoflurance and propofol	RCT	Lerman et al. (2009), Karanth et al. (2018)
	Sevoflurance and remifentanil	RCT	Joo et al. (2001), Lerman et al. (2009), Goo et al. (2017)
	Sevoflurance and dexmedetomidine	RCT	He et al. (2014), Wei et al. (2017)
	Propofol (or etomidate) and dexmedetomidine	RCT	Hanci et al. (2010), Bollucuoglu et al. (2013)
	Propofol and opioids	RCT	Akan et al. (2025), Hanci et al. (2010), Wei et al. (2015), Trabold et al. (2004), Erhan et al. (2003), Batra et al. (2004), Wei et al. (2011)
	Airway surface anesthesia	RCT	Rahmat Ameen Noorazyze et al. (2022), Zhang et al. (2023)
2. LMA Insertion	Not require complete elimination of vocal cord tension or pharyngeal muscle activity	RCT	Rahmat Ameen Noorazyze et al. (2022), Totonchi et al. (2022), Tang et al. (2023), Tulgar et al. (2017), Duman et al. (2001), van Vlymen et al. (2000)
Surgical requirements (Exposure)			
​	Phased titration of NMBAs	Observational	​
	Anesthetic drug combinations	RCT	Kang et al. (2017), Maidatsi et al. (2004), Wu et al. (2023), Paek et al. (2009)
	Combined epidural or spinal anesthesia	RCT	Munakata et al. (2004), Sahin et al. (2011), Suzuki et al. (2007), Hossain et al. (2012)

Abbreviations: RCT, randomized controlled trial.

## 6 Potential advantages and risks of minimizing or avoiding NMBAs

### 6.1 Advantages

Minimizing or avoiding NMBAs offers several significant advantages, particularly in the areas of postoperative recovery, minimizing complications, and suitability for day surgeries.

#### 6.1.1 Reduction of RNMB

Residual neuromuscular blockade (RNMB) is a common problem after anesthesia, particularly when neuromuscular blockade is not adequately monitored or reversed. Research indicates that RNMB is closely associated with postoperative respiratory complications, hypoxemia, and delayed recovery (Kirmeier et al., 2019). Reported incidences of RNMB (commonly defined as a TOF ratio <0.9) vary across studies but may be substantial, reaching approximately 65% in some multicenter reports (Frenkel and Lien, 2024; Saager et al., 2019). NMBA-sparing strategies guided by objective monitoring, and NMBA-free approaches in selected cases, may reduce the risk of RNMB, thereby minimizing the need for respiratory support and speeding up recovery. However, in settings where optimal intubating conditions are critical (e.g., rapid sequence induction or aspiration risk), NMBA-free or NMBA-sparing approaches may not be appropriate. Thus, these approaches should be individualized and balanced against first-pass success and airway safety.

#### 6.1.2 Accelerated recovery

The use of NMBAs may prolong the recovery phase, as these drugs continue to exert effects postoperatively. In selected patients, NMBA-free or NMBA-sparing approaches may facilitate shorter recovery times and earlier awakening. However, such strategies should be individualized to ensure that recovery benefits do not come at the expense of airway safety.

#### 6.1.3 Application in day surgery

In day surgery, minimizing or avoiding NMBAs offers significant advantages. Day surgeries require rapid postoperative recovery, enabling patients to safely discharge on the same day. By minimizing NMBAs usage, postoperative recovery is expedited, and the occurrence of complications (Kirmeier et al., 2019), including respiratory issues, is minimized, thereby enhancing patient comfort and improving hospital efficiency (Schreiber, 2014; Meistelman et al., 2019).

### 6.2 Risks

Despite the clear advantages of minimizing or avoiding NMBAs in certain settings, there are also risks associated with this strategy, particularly in terms of airway management failure, inadequate surgical exposure, and reliance on alternative medications.

#### 6.2.1 Airway intubation failure

Airway intubation is a high-risk procedure, especially in cases where muscle relaxation is insufficient. Excessive reduction or complete avoidance of NMBAs during airway management may lead to suboptimal conditions for intubation, increasing the risk of failure (Lundstrøm et al., 2018; Sneyd and O'Sullivan, 2010; Lundstrøm et al., 2009). For instance, in patients with complex anatomical conditions, minimizing NMBAs could impair airway visualization, thereby affecting the success of intubation. Therefore, attempts to minimize or avoid NMBAs should not compromise first-pass intubation conditions, adequate ventilation, or airway protection, particularly in high-risk or complex airway scenarios.

#### 6.2.2 Inadequate surgical exposure

While many surgeries can benefit from reduced neuromuscular blockade to enable faster recovery and fewer complications, some procedures—especially those requiring profound muscle relaxation, such as laparoscopic or thoracic surgeries—may face inadequate surgical exposure if muscle relaxants are reduced. In such cases, anesthesiologists must balance the reduction of muscle relaxants with ensuring sufficient surgical visibility and access. When inadequate exposure increases operative difficulty or operative time, the potential harms may outweigh recovery benefits, representing an important safety boundary for NMBA minimization.

#### 6.2.3 Need for alternative anesthetic medications

Minimizing or avoiding the use of NMBAs often requires the use of alternative medications, such as higher doses of opioids or inhalational anesthetics. These drugs may cause adverse effects like hypotension, especially when used in high doses or for prolonged periods. However, the feasibility and safety of NMBA-free or NMBA-sparing airway management are highly dependent on patient selection, airway characteristics, and clinical expertise, and the current evidence is largely derived from selected low-risk populations and specific procedural contexts. These advantages and their associated risks are summarized in Table 3.

**TABLE 3:** Advantages and Risks of minimizing or avoiding NMBAs.

Advantages	Risks	References
Reduction of residual neuromuscular blockade	Airway intubation failure	Lundstrøm et al. (2018), Sneyd and O'Sullivan (2010), Lundstrøm et al. (2009)
Accelerated recovery, reduction of postoperative Pulmonary complications	Inadequate surgical exposure	Cammu (2020), Frenkel and Lien (2024)
Suitable for day surgery	Dependence on alternative anesthetic medications	Schreiber (2014), Meistelman et al. (2019)

## 7 Re-evaluation of strategies in the sugammadex era

Sugammadex (Ravindranath et al., 2025; Linn and Renew, 2025) is a novel NMBA antagonist that can rapidly reverse the effects of non-depolarizing NMBAs, with the goal of achieving “no muscle relaxation” or “reduced residual muscle relaxation” as quickly as possible after surgery. The introduction of Sugammadex has had a profound impact on the use and management of NMBAs in anesthesiology. However, its introduction does not imply that NMBAs can be used indiscriminately without regard to dosage during general anesthesia (Todd and Kopman, 2023). Instead, it provides a new perspective on strategies to reduce or avoid the use of these agents.

### 7.1 Balancing “minimizing muscle relaxants” with “rapid reversal” in the context of sugammadex

The rapid reversal effect of Sugammadex enables anesthesiologists to quickly restore respiratory and motor functions postoperatively or when necessary. However, Sugammadex does not eliminate all risks associated with NMBAs (Honing et al., 2019). First, in cases of excessive use of NMBAs, Sugammadex may not fully antagonize their effects, particularly in the context of high doses or prolonged administration (Kotake et al., 2013). Secondly, the duration of Sugammadex’s reversal effect is limited, and excessive doses may increase the risk of respiratory depression after reversal (Renew and Linn, 2025; Ito et al., 2019). Despite these considerations, Sugammadex provides a safeguard for minimizing or avoiding NMBAs use, making low doses of NMBAs combined with rapid reversal a feasible option.

For patients who require some degree of muscle relaxation, using lower doses of NMBAs and rapidly reversing their effects with Sugammadex provides an effective balancing strategy. This approach can reduce the total amount of muscle relaxants used, thus shortening postoperative recovery times while ensuring patient safety. In clinical practice, two strategies are commonly considered: NMBA-free and NMBA-sparing with sugammadex reversal. The choice between these approaches should be individualized based on surgical requirements, patient risk, availability of neuromuscular monitoring, and institutional resources.

### 7.2 Limitations and clinical applications of sugammadex

Despite its clinical advantages, sugammadex is not a universal solution. It is effective only against non-depolarizing NMBAs (e.g., rocuronium) and not against depolarizing agents like succinylcholine. Moreover, its relatively high cost may limit widespread use, particularly in resource-limited settings (Hawkins et al., 2019). From a pharmacoeconomic perspective, this higher acquisition cost may be partially offset in selected settings by reductions in residual blockade-related events, unplanned ventilatory support, and recovery-room delays, thereby improving workflow efficiency. However, the magnitude of these benefits is context-dependent, influenced by case mix, local pricing, and monitoring practices.

Importantly, sugammadex availability does not eliminate the need for quantitative neuromuscular monitoring. Monitoring remains essential when deep or repeated neuromuscular blockade is used and for confirming adequate recovery before extubation. Therefore, in the sugammadex era, optimized NMBA use guided by objective monitoring may represent a safer and more reliable strategy than unmonitored NMBA avoidance.

## 8 Patient and surgical procedure selection

This section is intended to define practical “safety boundaries” for NMBA-free and NMBA-sparing strategies, emphasizing that these approaches should not be considered default options and must be guided by patient risk, surgical requirements, and available monitoring/reversal resources. Strategies to minimize or avoid the use of NMBAs are not suitable for all patients and surgical procedures. They depend on individualized patient assessment and the specific requirements of the surgical procedure (Döcker and Walther, 2012). Overall, NMBA-free anesthesia should not be interpreted as a default or universally superior anesthetic strategy; individualized decision-making remains essential based on patient risk, surgical requirements, and available monitoring and reversal resources. Below are the types of patients and procedures that are appropriate or not appropriate for this strategy.

### 8.1 Patients and surgical procedures suitable for minimizing or avoiding muscle relaxants

- Expected ease of airway management: Normal airway structure without significant anatomical abnormalities.
- Low risk of aspiration: No history of gastroesophageal reflux or gastrointestinal disorders.
- Superficial or limb surgeries: Such as skin excision, burn grafting, thyroid surgery, breast surgery, lower limb and upper limb surgeries, and urological endoscopic surgeries. These procedures generally do not involve complex anatomical operations or high requirements for muscle relaxation.
- Other special scenarios: Day surgery (Bettelli, 2006), procedures requiring intraoperative neurophysiological monitoring, intraoperative awakening, or procedures where spontaneous breathing must be preserved, such as tubeless thoracic surgeries.

### 8.2 Patients and surgical procedures unsuitable for minimizing muscle relaxants

• Difficult airway patients (including pediatric patients at risk of unanticipated difficult airway): Such as obese patients or those with upper airway anatomical abnormalities. For these patients, it may be necessary to preserve spontaneous breathing during induction to avoid the use of NMBAs. However, once adequate ventilation is confirmed, the use of neuromuscular blocking agents may be considered to facilitate tracheal intubation and improve first-pass success, and a clearly defined rescue strategy should be in place (Black et al., 2015).
• Procedures requiring deep muscle relaxation: Such as extensive open or laparoscopic abdominal surgeries, open or laparoscopic thoracic surgeries, and orthopedic surgeries. Major orthopedic procedures like spinal surgery, hip replacement, and surgeries involving the oral and pharyngeal areas, such as tonsillectomy, fall into this category.
• Procedures requiring absolute immobility: Such as microsurgical neurosurgery and interventional procedures (e.g., intracranial aneurysm embolization).

## 9 Limitations

Although the strategy of minimizing or avoiding NMBAs has made some progress in its application during general anesthesia for surgical procedures, several limitations should be acknowledged. This review was conducted as a structured narrative review; therefore, no formal risk-of-bias assessment or evidence grading framework was applied, and no quantitative synthesis was performed. Moreover, the available evidence is heterogeneous in study design, patient selection, anesthetic techniques, and outcome definitions, and much of the data derive from selected low-risk elective populations, which may limit generalizability to high-risk or complex clinical settings. Existing clinical studies remain limited, particularly across different patient populations and surgical types. There remains a paucity of data on special populations, including elderly, obese, and obstructive sleep apnea (OSA) patients. Furthermore, only English-language literature was included, which may have restricted the inclusion of relevant studies published in other languages.

In addition, implementation challenges remain. Optimizing drug combinations, titrating anesthetic depth, and integrating modern airway management tools require experience and structured guidance. Unlike depth-of-anesthesia monitoring (e.g., BIS), objective neuromuscular monitoring is not yet routinely implemented in many settings, which may affect consistent and safe adoption of NMBA-free and NMBA-sparing strategies.

## 10 Future research directions

Although the strategy of minimizing or avoiding NMBAs has shown some success in clinical practice, several issues remain unresolved. Future research should focus on the following areas.

### 10.1 Further clinical studies: non-inferiority and optimization of drug combinations

While existing studies suggest that minimal or no NMBAs strategies are effective in certain patient populations, there is a lack of non-inferiority studies to verify whether this strategy is universally applicable to all patients. Future research should focus on optimizing drug combinations, exploring which combinations provide the best airway management outcomes across different surgeries, while minimizing the over-reliance on NMBAs.

### 10.2 Applications in specific populations: elderly, obese, and OSA patients

For elderly patients, obese individuals, and those with OSA, minimizing NMBAs use may present distinct risks and benefits. Future studies should place greater emphasis on the effectiveness and safety of minimizing or avoiding NMBAs in these high-risk populations.

## 11 Conclusion

The strategy of minimizing or avoiding NMBAs presents a new direction in general anesthesia. Through a judicious combination of pharmacological agents, the integration of various anesthetic techniques, modulation of anesthetic depth, and the use of novel airway management tools, anesthesiologists can reduce reliance on NMBAs while ensuring airway safety and meeting surgical requirements. This approach may help decrease residual muscle relaxants and postoperative pulmonary complications, ultimately promoting faster recovery for patients in selected settings, particularly when combined with appropriate patient selection and objective neuromuscular monitoring.

However, this strategy is not suitable for all patients and surgical procedures. Individualized anesthetic management remains the key to successful implementation. Future research should focus on optimizing drug combinations, validating non-inferiority, and exploring applications for specific patient populations. Large-scale, multi-center clinical studies are particularly needed to assess not only the practical implementation strategies but also the effects of this approach on postoperative pulmonary complications, length of hospital stay, long-term functional recovery, and healthcare economics (Grabitz et al., 2019). These studies will help define the optimal clinical scenarios and implementation guidelines for this strategy. Ultimately, evidence-based research will contribute to the development of clinical practice guidelines, further advancing perioperative management.
